# Mechanistic Studies on the Absorption-Enhancing Effects of Gemini Surfactant on the Intestinal Absorption of Poorly Absorbed Hydrophilic Drugs in Rats

**DOI:** 10.3390/pharmaceutics11040170

**Published:** 2019-04-07

**Authors:** Tammam Alama, Kosuke Kusamori, Masaki Morishita, Hidemasa Katsumi, Toshiyasu Sakane, Akira Yamamoto

**Affiliations:** 1 Department of Biopharmaceutics, Kyoto Pharmaceutical University, Misasagi, Yamashina-Ku, Kyoto 607-8414, Japan; abowaleed87@gmail.com (T.A.); morishita@mb.kyoto-phu.ac.jp (M.M.); hkatsumi@mb.kyoto-phu.ac.jp (H.K.); 2 Laboratory of Biopharmaceutics, Faculty of Pharmaceutical Sciences, Tokyo University of Science, 2641 Yamazaki, Noda, Chiba 278-8510, Japan; kusamori@rs.tus.ac.jp; 3 Department of Pharmaceutical Technology, Kobe Pharmaceutical University, Higashinada-ku, Kobe 658-8558, Japan; sakane@kobepharma-u.ac.jp

**Keywords:** absorption enhancer, gemini surfactant, intestinal absorption, poorly absorbed drug, Caco-2 cells

## Abstract

Generally, the use of absorption enhancers might be the most effective approaches to ameliorate the enteric absorption of poorly absorbed substances. Among numerous absorption enhancers, we already reported that a gemini surfactant, sodium dilauramidoglutamide lysine (SLG-30) with two hydrophobic and two hydrophilic moieties, is a novel and promising adjuvant with a high potency in improving the absorption safely. Here, we examined and elucidated the absorption-improving mechanisms of SLG-30 in the enteric absorption of substances. SLG-30 increased the intestinal absorption of 5(6)-carboxyfluorescein (CF) to a greater level than the typical absorption enhancers, including sodium glycocholate and sodium laurate, as evaluated by an *in situ* closed-loop method. Furthermore, SLG-30 significantly lowered the fluorescence anisotropy of dansyl chloride (DNS-Cl), suggesting that it might increase the fluidity of protein sections in the intestinal cell membranes. Moreover, SLG-30 significantly lowered the transepithelial-electrical resistance (TEER) values of Caco-2 cells, suggesting that it might open the tight junctions (TJs) between the enteric epithelial cells. Additionally, the levels of claudin-1 and claudin-4 expression decreased in the presence of SLG-30. These outcomes propose that SLG-30 might improve the enteric transport of poorly absorbed substances through both transcellular and paracellular routes.

## 1. Introduction

The enteric epithelium represents a physical barrier for the intestinal absorption of most substances to the systemic-circulation. It is well-established that the enteric epithelium acts as an impervious barrier against many pathogens and toxins through its complicated structure, although it can permit the intestinal absorption of nutrients [1]. The most important barrier against the permeability of drugs is the brush-border membranes of the enteric epithelial cells, which can restrict the passage of drugs through cells into the systemic circulation. The other important barrier is the tight junction between the adjacent cells, which is an aqueous route for compounds of molecular weight less than 500 Da [2,3,4]. These two barriers are considered great hurdles when some drugs are transported across the intestinal epithelial cells, assuming that drugs could survive the harsh environment and the decomposition in the gastrointestinal tract [5,6]. Therefore, the major routes for drugs to be absorbed by the intestinal cells are the transcellular and paracellular routes [7,8,9]. This could be a serious problem for a wide range of newly discovered and synthesized drugs, especially peptide and protein drugs, which also suffer from enzymatic deterioration [10] in the gastrointestinal tract before reaching the enteric cell membrane.

A variety of systems have been tested to improve the enteric absorption of poorly absorbed substances [1]. Of all these approaches, the use of absorption enhancers is a promising way to improve the enteric absorption of poorly absorbed substances [11,12,13,14,15,16,17,18,19,20]. It was reported that typical absorption enhancers involving medium chain fatty acids (e.g., sodium laurate, sodium caprate, and sodium caprylate) could improve the absorption of poorly absorbed substances such as antibiotics, peptide and protein drugs, and bisphosphonates [11,12,13,14,15,16,17,18,19,20]. However, most of the typical absorption-enhancers usually prompt irritation along with damage to enteric membranes.

Gemini surfactants are a new type of absorption enhancers with a rather different structure than the typical ones [21]. The structure is composed of two hydrophilic heads connected with a linker and two hydrophobic tails. This structure makes the CMC values of gemini-type surfactants lower than the CMC values of typical surfactants with an equal chain length [21]. However, the effects of gemini surfactants on the enteric absorption of substances and how they improve absorption are seldom studied. 

**Figure 1:**
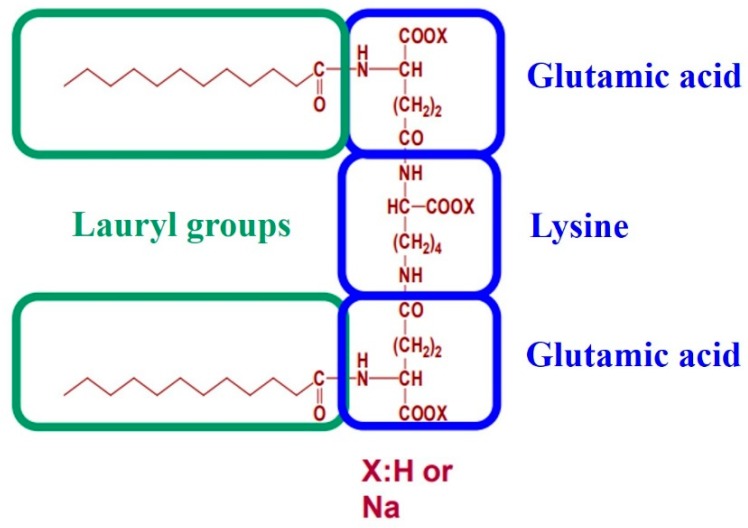
The chemical structure of a gemini surfactant, SLG-30. The hydrophilic moiety of this surfactant has two derivatives of amino acid, i.e., two glutamic acid moieties, linked through a lysine spacer, while the hydrophobic moieties are lauric acid.

We previously reported SLG-30, a gemini surfactant, increased the enteric absorption of hydrophilic poorly absorbed substances [22]. As shown in Figure 1, the hydrophilic heads of SLG-30 have two amino acid derivatives, i.e., two glutamic acid moieties, linked through a lysine spacer, and two hydrophobic chains of twelve carbons each [23]. We showed that SLG-30 might be safe *in vitro* and *in vivo* and may be a very efficient absorption enhancer, which can possibly be used as an adjuvant in oral formulations for increasing the enteric absorption of poorly absorbed hydrophilic substances. However, we did not compare the effectiveness of SLG-30 with that of typical absorption enhancers regarding the enteric absorption of substances. Moreover, the absorption-enhancing mechanisms of SLG-30 in improving the enteric absorption of poorly absorbed substances were not clearly understood. 

The purpose of this study was to study the difference between SLG-30, a gemini surfactant, with typical absorption enhancers, including sodium glycocholate and sodium laurate, regarding absorption-enhancing performance. Furthermore, we also examined and clarified the possible absorption-enhancing mechanisms of SLG-30 on the enteric absorption of substances. 

## 2. Materials and Methods

### 2.1. Materials

Male Wistar rats, weighing 250–300 g, were purchased from SLC, Inc. (Hamamatsu, Shizuoka, Japan). SLG-30 was kindly supplied by Asahi Kasei Chemicals Co. (Tokyo, Japan). CF was obtained from Eastman Kodak Co. (Rochester, NY, USA). LDH-Cytotoxicity Test Wako, sodium glycocholate (NaGC), sodium laurate, and tma-DPH (1-(4-(trimethylamino)phenyl)-6-phenylhexa-1,3,5-hexatriene-p-toluenesulfonate) were purchased from Wako Pure Chemical Industries, Ltd. (Osaka, Japan). DPH (1,6-diphenyl-1,3,5-hexatriene) and Hank’s balanced salt solution (HBSS) were purchased from Sigma-Aldrich Chemical Co. (St. Louis, MO, USA). Dansyl chloride (DNS-Cl) was purchased from Santa Cruz Biotechnology, Inc. (Dallas, Texas, USA). Anti-claudin-1, anti-β-actin (rabbit monoclonal antibodies), and goat anti-rabbit IgG HRP-linked antibodies were purchased from Cell Signaling Technology^®^ (Danvers, MA, USA). Anti-claudin-4 (mouse monoclonal antibodies) and rabbit anti-mouse IgG HRP-linked antibodies were purchased from Invitrogen™ (Carlsbad, CA, USA). Chemi-Lumi One Ultra kit, Dulbecco’s modified Eagle medium (DMEM) with 4500 mg/L glucose, nonessential amino acids (MEM–NEAA), antibiotic–antimycotic mixture stock (10,000 U/mL penicillin, 10,000 µg/mL streptomycin, and 25 µg/mL amphotericin B in 0.85% sodium chloride), and 0.25% trypsin-1 mM EDTA solution were purchased from Nacalai Tesque Inc. (Kyoto, Japan). Human colon adenocarcinoma-derived Caco-2 cell line was purchased from Dainippon Sumitomo Pharma Co., Ltd. (Osaka, Japan). Fetal bovine serum (FBS) was purchased from Gibco^®^ Life Technologies (Grand Island, NE, USA). All other reagents used in the experiments were of analytical grade. 

### 2.2. In Vivo Enteric Absorption Studies

The enteric absorption of CF was examined by an *in situ* closed-loop method, as reported previously [22,24]. The experiments were carried out in accordance with the guidelines of the Animal Ethics Committee at Kyoto Pharmaceutical University. The protocols were approved by this Committee (permit number: 16-12-069, permit date: 14 April 2016). The rats were starved overnight for approx. 16 h pre-dosing, but water was freely available. After inducing anesthesia with sodium pentobarbital which was administered intraperitoneally at a dose of 32 mg/kg body weight, the rats were placed under a heat lamp to maintain a body temperature at around 37 °C, and the intestines were exposed using a midline-abdominal incision. After the bile duct was ligated, the intestines were washed with phosphate buffered saline (PBS, pH 7.4), and the remaining buffer solution was expelled with air. Enteric cannulation was performed at both ends using polyethylene tubing, and the distal parts of the small intestines were clipped by forceps. The dosing solutions (3 mL) with or without absorption enhancers kept at 37 °C, were directly introduced into the lumen of the enteric loop through a cannulated opening in the proximal part of the small enteric loop, which was then closed by clipping with another forceps. The jugular vein was exposed, and approx. 0.3 mL of blood was collected via a direct puncture into heparinized syringes at predetermined time intervals up to 240 min. The samples were immediately centrifuged at 12,000 rpm (15,000× *g*) for a period of 5 min to obtain the plasma fraction, which was stored on ice for further analysis. CF fluorescence signals in these plasma samples were detected after the treatment with the same volume of acetonitrile using a fluorescence spectrophotometer, Powerscan1 HT supplied by BioTek Instruments (Winooski, VT, USA) at an excitation wavelength of 485 nm and an emission wavelength of 535 nm. There was no significant background fluorescence in the plasma when the fluorescence intensities of CF were determined in this study. The peak drug concentrations (C_max_) and the time to reach the peak drug concentrations (T_max_) in the plasma were directly determined from the plasma concentration–time profiles. The area under the curve (AUC) was calculated by the trapezoidal method from pre-dose (time zero) till the administration of the final sample. The absorption enhancement ratios of CF, with or without absorption enhancers, were calculated as follows:

Absorption enhancement ratio = AUC_with enhancer_/AUC_control without enhancer_

### 2.3. Preparation of Brush Border Membrane Vesicles (BBMVs)

To prepare BBMVs, we followed the methods as reported previously [25,26,27] with a slight modification. Briefly, an *in situ* small intestinal loop was prepared in each rat, as mentioned above, and then, PBS (pH 7.4) was administered into the loop. The fat was trimmed off from the small intestine and mesentery. Then, the whole small intestine was soaked in ice-cold PBS (pH 7.4). The small intestine was divided into 10 cm segments. Mucosa was scraped out with a slide glass from each of these segments and used for subsequent studies. BBMVs were prepared by the divalent cation precipitation method using MgCl_2_ in the presence of ethylenebis (oxyethylenenitrilo) tetraacetic acid (EGTA) [25,26,27]. Briefly, the collected mucosa was homogenized in a buffer containing mannitol (300 mM), EGTA (5 mM), and Tris (pH 7.4) (12 mM) by using a tissue homogenizer. An aqueous solution of 10 mM magnesium chloride was added to the homogenate. The homogenate was centrifuged at 3000× *g* for 15 min. The supernatant was then centrifuged at 32,000× *g* for 30 min. The pellet was resuspended in a buffer containing mannitol (300 mM), EGTA (5 mM), and Tris (pH 7.4) (12 mM) by using a 26-G needle. The protein concentration was determined by the BCA method using bovine serum albumin as a standard, and the final concentration was adjusted to 1 mg/ml in each Eppendorf tube. The samples were frozen by liquid nitrogen and kept at −80 °C for further studies.

### 2.4. Measurement of Membrane Fluidity by Fluorescence Polarization

The BBMVs (100 μg protein) were incubated with 1 μM DPH, with 0.5 μM tma-DPH or with 5 μM DNS-Cl in HEPES-Tris buffer (25 mM HEPES, 5.4 mM KCl, 1.8 mM CaCl_2_, 0.8 mM MgSO_4_, 140 mM NaCl, 5 mM glucose, pH 7.4 adjusted by 1 M Tris) in the dark at 37 °C for 30 min [25,28]. Then, various concentrations (0.1% *v*/*v*, 0.25% *v*/*v*, 0.5% *v*/*v*, and 1.0% *v*/*v*) of SLG-30 were added. Then, the samples were incubated in the dark for 1 min at 37 °C. For the control group, the same procedure was carried out by adding the HEPES-Tris buffer only. The fluorescence intensities and the steady-state polarization of fluorescence expressed as the fluorescence anisotropy, r, of the labeled membrane vesicles were measured at 37 °C. The excitation and emission wavelengths were λ_ex_ = 360 nm, and λ_em_ = 430 nm for DPH and tma-DPH and λ_ex_ = 380 nm and λ_em_ = 480 nm for DNS-Cl. The measurements were carried out by using a Hitachi Spectrofluorometer (F-2000 Spectrofluorometer, Hitachi Seisakusho Corp, Yokohama, Japan) equipped with a polarizer set.

The fluorescence anisotropy (r) was calculated using the following equation:

$$r=\frac{I_{V}-I_{H}}{I_{V}+2I_{H}}$$

where *I*_V_ and *I*_H_ respectively represent the perpendicular and parallel fluorescence intensities to the polarized excitation plane [29].

### 2.5. Measurement of Transepithelial Electrical Resistance (TEER) and the Transport of CF Using Caco-2 cell Monolayers

Caco-2 cells (passage 45) were cultured in 175-cm^2^ culture flask (Thermo Fisher Scientific™, Waltham, MA, USA). The culture medium consisted of DMEM containing 10% FBS, 0.1 mM MEM–NEAA, 2 mM glutamine, 100 U/mL penicillin, and 100 µg/mL streptomycin [30]. Cells were cultured in a humidified atmosphere of 5% CO_2_ at 37 °C. The culture medium was changed every two days. When the cultured Caco-2 cells became sub-confluent, they were seeded onto 12-mm Transwell^®^ with 0.4-µm pore polycarbonate membrane inserts (Corning Inc., New York, NY, USA) at a density of 1 × 10^5^ cells/insert. The transepithelial transport studies were performed when the transepithelial electrical resistance (TEER) values were more than 500 Ω·cm^2^ (i.e., after 21 days) [17,18]. Briefly, after removing the incubation medium by aspiration, the apical and basal sides were washed thrice by Hank’s Balanced Salt (HBSS) solution (pH 7.4). The cells were incubated in HBSS (pH 6.0) for the apical side and HBSS (pH 7.4) for the basal side for 20 min at 37 °C. HBSS supplemented with 1 mg/mL glucose was used in all cell culture experiments. After removing the washing solution by aspiration, 500 µL of 10 µM CF in a HBSS (pH 6.0) solution with or without various concentrations (0.001% *v*/*v*, 0.01% *v*/*v*, 0.025% *v*/*v*, 0.05% *v*/*v*, and 0.1% *v*/*v*) of SLG-30, 0.1% (*w*/*v*) and 1% (*w*/*v*) NaGC or 0.05% (*w*/*v*) sodium laurate were added to the apical side at zero time, whereas precisely 1500 µL of HBSS (pH 7.4) was added to the basal side at 37 °C. The cells were kept at 37 °C and 5% CO_2_ and were continuously agitated on a shaker during the transport experiments.

The TEER values were measured by Millicell^®^ (ERS-2 Volt-Ohm Meter, Billerica, MA, USA) at the predetermined times up to 24 h, and the initial values were considered as 100%. The samples from the basal side were withdrawn at predetermined time points up to 24 h. The samples were replaced with an equal volume of HBSS (pH 7.4). CF was determined as previously mentioned. The apparent permeability coefficients *P_app_* (cm/s) for the transported CF was determined by the following equation:

$$P_{app}=\frac{{dX}_{R}}{dt}\times \frac{1}{A}\times \frac{1}{C_{0}}$$

where *P_app_* is the apparent permeability coefficient in centimeters per second, *X_R_* is the amount of CF in moles in the receptor side, $\frac{{dX}_{R}}{dt}$ is the flux across the monolayer, *A* is the diffusion area (i.e., in square centimeters), and *C*_0_ is the initial concentration of CF in the donor side in moles per milliliter.

### 2.6. Assessment of Caco-2 Cell Damage

After the transport studies were over, the solution on the apical side of each well was collected. Cell injury caused by SLG-30 was determined by tracking the LDH release [31,32]. The cell precipitate and the monolayers left on the filters were solubilized with 1% (*v*/*v*) Triton X-100. The solutions from apical sides and the lysates were centrifuged for 7 min at 200× *g* at a temperature of 4 °C. The activities of LDH in the supernatant were measured. (LDH_release_) stands for the activity of the LDH released into the apical side in the existence of SLG-30. (LDH_cell_) stands for the activity of the LDH obtained from the cell lysate. LDH_released_% was calculated by the following equation:

LDH_release_% = LDH_release_ × 100/(LDH_release_ + LDH_cells_)

### 2.7. Western Blotting

Western blotting was performed by the method described previously with slight modifications [33,34]. Three male Wistar rats weighing 250 g were prepared to perform an *in situ* closed-loop method on the small intestine, as mentioned above. A control study, a treatment study, and a pretreatment study were conducted on the first, second, and third rat, respectively. For the control study, only PBS solution (pH 7.4) was administered into the small intestine, and then, the rat was sacrificed, while for the treatment study, SLG-30 was administered for 1 h, and then, the rat was sacrificed without washing the SLG-30 out. The pretreatment study was performed by administrating SLG-30 for 1 h then washing it out with PBS solution (pH 7.4), followed by sacrificing the rat after 4 h. The small intestine (60 cm) of three rats was taken and treated, as mentioned above, for extracting the BBMVs. The total protein amount of each sample was adjusted to 30 μg.

The expression levels of proteins from the claudin family, in the homogenate of the small intestine membrane, were evaluated by western blotting. Briefly, equal amounts of protein samples (30 μg protein) were mixed with an SDS buffer solution and electrophoretically separated on SDS-polyacrylamide (15%) gels. The separated proteins were transferred to a polyvinylidene difluoride (PVDF) membrane. After blocking in 5% skim milk in Tris-buffered saline (pH 7.4) for 1 h at room temperature, the PVDF membrane was incubated overnight in a blocking buffer with diluted (1:1000) monoclonal antibodies for claudin-1, claudin-4, and β-actin at 4 °C. Subsequently, the PVDF membrane was washed three times using Tris-buffered saline containing 0.05% Tween 20 (TTBS), followed by incubation with peroxidase-conjugated anti-rabbit IgG antibody for claudin-1 and β-actin, and with peroxidase-conjugated anti-rabbit IgG antibody for claudin-4 for 1 h at room temperature. The signals were visualized by luminescence imaging (Fujifilm Luminescent Image Analyzer LAS4000 System, Tokyo, Japan). The intensity of each signal was corrected using the values obtained from the β-actin bands, and the relative protein intensity was expressed as the fold change compared to the relative protein intensity in the normal group. 

### 2.8. Statistical Analysis

The results are expressed as the mean ± S.E. of at least three experiments. The statistical significance between groups was analyzed using Dunnett’s test; *p* < 0.05 was regarded to be significant. Significance levels are denoted (**) *p* < 0.01, (*) *p* < 0.05, and (n.s.) not significantly different. The number of experiments is indicated by *n*.

## 3. Results

### 3.1. In Vivo Comparative Study between SLG-30 and Typical Surfactants Including Sodium Glycocholate and Sodium Laurate

**Figure 2:**
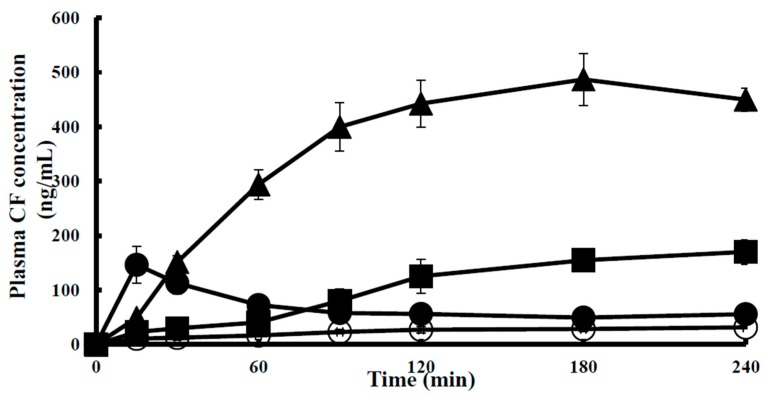
The profile of concentration vs. time course of 5(6)-carboxyfluorescein (CF) in the plasma after an enteric administration with SLG-30, a gemini surfactant, or typical absorption enhancers including sodium glycocholate and sodium laurate by an *in situ* closed-loop method. The results are expressed as the mean ± S.E. (*n* = 3). Keys: (◯) Control, (▲) 0.5% (*v*/*v*) SLG-30, (●) 0.5% (*w*/*v*) sodium laurate, and (■) 1% (*w*/*v*) sodium glycocholate.

First, the effects of 0.5% (*v*/*v*) SLG-30 and 1% (*w*/*v*) sodium glycocholate (NaGC) on the enteric absorption of CF were examined by an *in situ* closed-loop method, and the effectiveness of SLG-30 was compared with that of NaGC (a typical absorption enhancer). As Figure 2 shows, the enteric absorption of CF was significantly improved in the existence of 0.5% (*v*/*v*) SLG-30, and the AUC_0→240_ of CF was 16 times higher than that of the control group, as opposed to 4.5 times in the presence of 1% (*w*/*v*) NaGC. The absorption improvement of CF in the existence of 0.5% (*v*/*v*) SLG-30 was about 3.5 times higher than that in the existence of 1% (*w*/*v*) NaGC (Table 1). As shown in Figure 1, SLG-30 contains two moieties of fatty acid (lauric acid). Therefore, in order to clarify whether SLG-30 is effective as a whole compound or whether the effectiveness is related to lauric acid, a degradation product of SLG-30, we also studied the absorption improvement effect of 0.5% (*w*/*v*) sodium laurate in the small intestines by the *in situ* closed-loop method. We found that the T_max_ of CF in the case of sodium laurate-treated samples is approximately 15 min. Thereafter, the concentrations of CF decreased quickly and continuously until the end of the experiment. Contrarily, SLG-30 greatly increased the concentrations of CF until T_max_ (180 min), followed by a decrease, as revealed in Figure 2 and Table 1. The absorption improvement ratio of CF for SLG-30 was approximately 6 times higher than that of sodium laurate. 

### 3.2. Effects of SLG-30 on the Membrane Fluidity of Small Intestines in Rats

**Figure 3:**
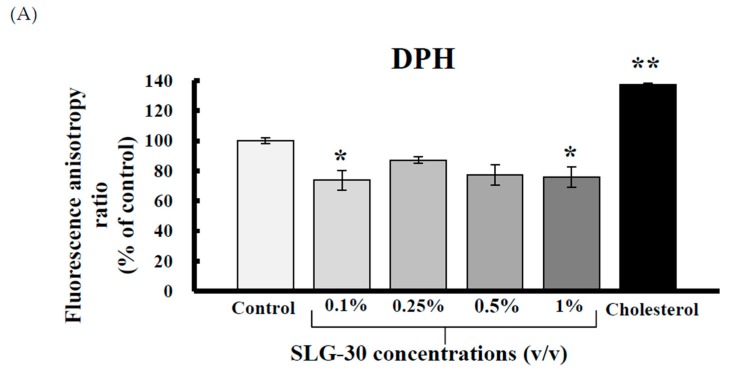

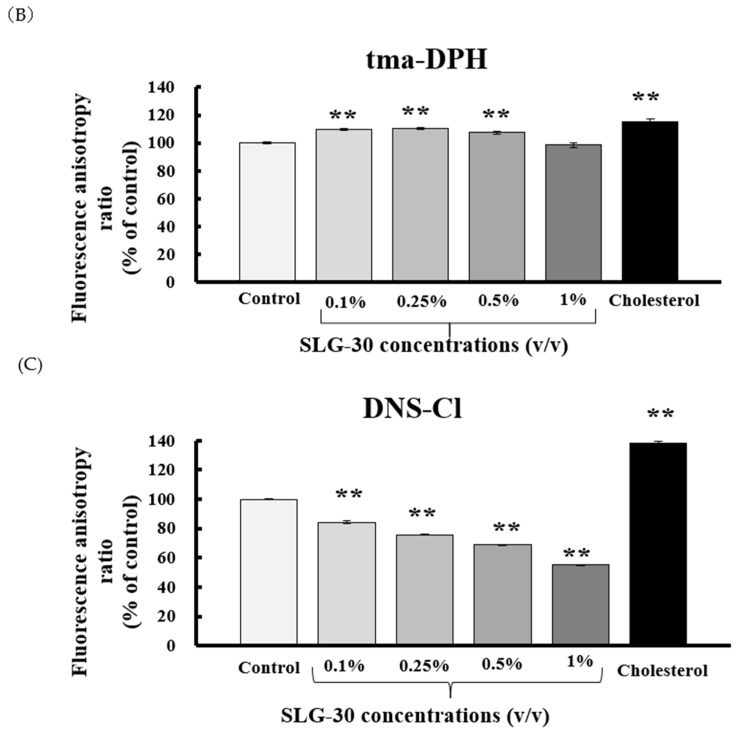
The effect of SLG-30 on the membrane fluidity of brush-border membrane vesicles (BBMVs). DPH (**A**), tma-DPH (**B**), and DNS-Cl (**C**) were used as fluorescein probes of inner lipid bilayers, outer lipid bilayers, and the protein portion of the enteric membranes. The absolute concentrations of DPH, tma-DPH, and DNS-Cl were 1 μM, 0.5 μM, and 5 μM, respectively. The results are expressed as the mean ± S.E. (*n* = 4). ** *p* < 0.01, n.s. not significantly different when compared with the control.

One of the important absorption-enhancing mechanisms of absorption enhancers is that they increase the membrane fluidity of the enteric membrane, thereby increasing the enteric absorption of poorly absorbed substances through a transcellular route. To examine the effect of SLG-30 on the enteric membrane fluidity, DPH, tma-DPH, and DNS-Cl, which can bind to different portions of the lipid bilayers (i.e., inner, outer, and protein portion, severally) were used as fluorescence probes in this study. Any decrease in the fluorescence anisotropy of these markers represents an increase in the membrane fluidity of BBMVs. Figure 3A–C shows that cholesterol, used as a positive control, increased the fluorescence anisotropy, which indicates a decrement in the membrane fluidity of BBMVs. Figure 3A shows that SLG-30 slightly decreased the fluorescence anisotropy of DPH compared to that of the control group. However, as Figure 3B shows, SLG-30 did not decrease, but significantly increased the tma-DPH fluorescence anisotropy. However, SLG-30 significantly reduced the fluorescence anisotropy of DNS-Cl in a dose dependent pattern (** *p* < 0.01 vs. control group) (Figure 3C). These findings propose that the membrane fluidity in the protein section of the enteric membranes could be significantly augmented by SLG-30, while it also slightly augmented the membrane fluidity in the inner section of the lipid bilayers.

### 3.3. Effect of SLG-30 on TEER and Transport of CF in Caco-2 Cell Monolayers

**Figure 4:**
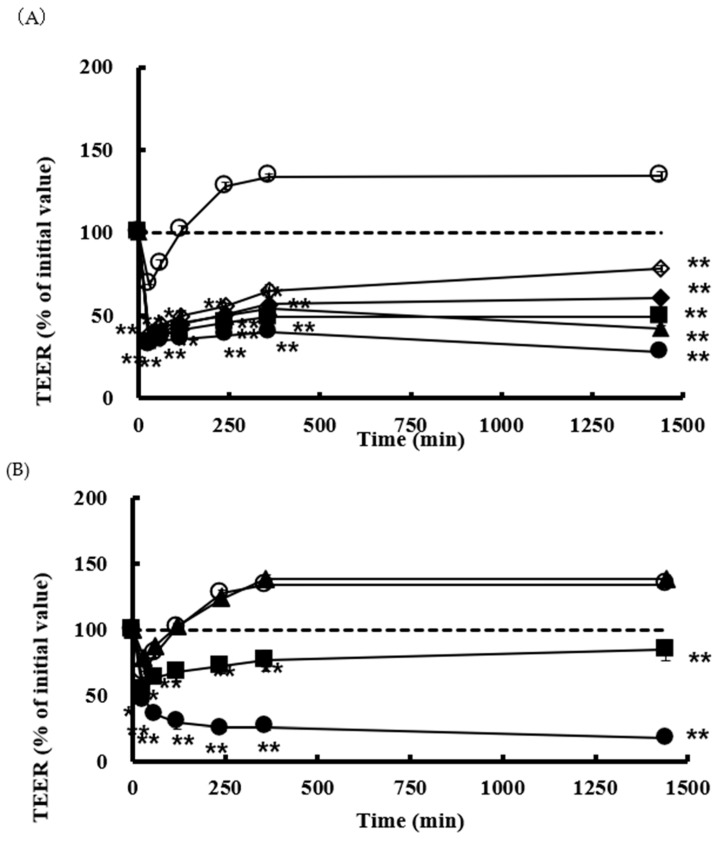
The TEER values in the existence of (**A**) SLG-30 and (**B**) sodium glycocholate and sodium laurate in Caco-2 cell monolayers. The results are expressed as the mean ± S.E. (*n* = 3). ** *p* < 0.01, n.s. not significantly different when compared with the control. Keys for Figure 4A: (◯) Control, (◇) 0.001% (*v*/*v*) SLG-30, (◆) 0.01% (*v*/*v*) SLG-30, (■) 0.025% (*v*/*v*) SLG-30, (▲) 0.05% (*v*/*v*) SLG-30, and (●) 0.1% (*v*/*v*) SLG-30. Keys for Figure 4B: (◯) Control, (▲) 0.1% (*w*/*v*) sodium glycocholate, (■) 1% (*w*/*v*) sodium glycocholate, and (●) 0.05% (*w*/*v*) sodium laurate.

**Figure 5:**
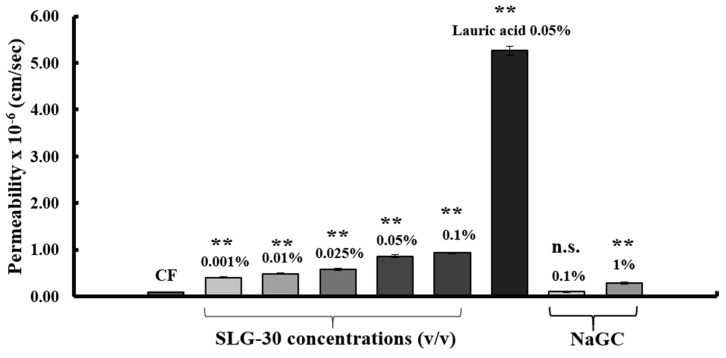
The permeability of CF in the presence of SLG-30, sodium glycocholate, and sodium laurate across Caco-2 cell monolayers: The results are expressed as the mean ± S.E. (*n* = 3). ** *p* < 0.01, n.s. not significantly different when compared with the control.

We also estimated the effects of SLG-30 on the TEER values and the transport of poorly absorbed substances through the paracellular route by using Caco-2 cell monolayers. CF solutions in HBSS with or without various concentrations of SLG-30 (i.e., 0.001% *v*/*v*, 0.01% *v*/*v*, 0.025% *v*/*v*, 0.05% *v*/*v*, and 0.1% *v*/*v*) were added to the apical side of the cells and incubated at 37 °C. As shown in Figure 4A, a significant decrease in the TEER values was noticed in the presence of SLG-30 at all concentrations studied, including the lowest concentration of 0.001% (*v*/*v*), in the first hour. The TEER values did not recover to the baseline (without removing SLG-30) for the higher concentrations, but they started to recover for the lower concentrations. In contrast to the TEER results, as shown in Figure 5, the transport of CF significantly increased after 24 h upon coadministration with SLG-30 at all concentrations. The enhancement ratios of the CF transport were 6, 7, 9, 11, and 12 times higher than the control group for SLG-30 concentrations of 0.001% (*v*/*v*), 0.01% (*v*/*v*), 0.025% (*v*/*v*), 0.05% (*v*/*v*), and 0.1% (*v*/*v*), respectively.

Parallel studies were performed with 0.1% (*w*/*v*) and 1% (*w*/*v*) NaGC and with 0.05% (*w*/*v*) sodium laurate. Figure 4B shows that TEER values were significantly reduced in the presence of 0.05% (*w*/*v*) sodium laurate without recovering to the baseline within the period of the experiment. In the case of 1% (*w*/*v*) NaGC, the TEER values significantly decreased then recovered to the baseline at the end of the experiment. Almost no effect was seen with 0.1% (*w*/*v*) NaGC on the TEER values. As shown in Figure 5, 0.05% (*w*/*v*) sodium laurate significantly increased (94 times) the transport of CF as compared to that observed in the control group; 1% (*w*/*v*) NaGC also significantly increased the transport of CF to a lesser extent than SLG-30 (i.e., 5 times higher than the control group). Further, 0.1% (*w*/*v*) NaGC had an insignificant effect on the transport of CF through Caco-2 cells (Figure 5).

### 3.4. Assessment of Caco-2 Cells Injury

**Figure 6:**
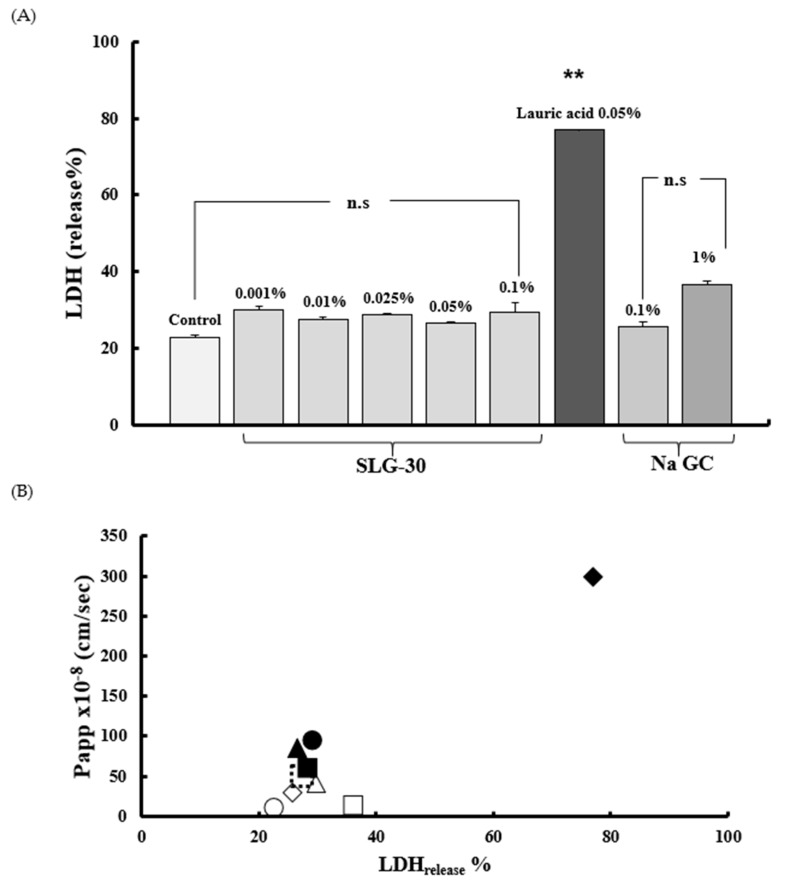
(**A**) The evaluation of the membrane toxicity of Caco-2 cells monolayers in the existence of SLG-30, sodium glycocholate, and sodium laurate and (**B**) the relationship between the effects of SLG-30 and typical absorption enhancers on the CF permeability across Caco-2 cells and their toxic effects: The results are expressed as the mean ± S.E. (*n* = 6). ** *p* < 0.01, n.s. not significantly different when compared with the control. Keys for Figure 6B: (◯) Control, (∆) 0.001% (*v*/*v*) SLG-30, (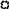) 0.01% (*v*/*v*) SLG-30, (■) 0.025% (*v*/*v*) SLG-30, (▲) 0.05% (*v*/*v*) SLG-30, (●) 0.1% (*v*/*v*) SLG-30. (◆) 0.1% (*w*/*v*) sodium glycocholate, (□) 1% (*w*/*v*) sodium glycocholate, and (◆) 0.05% (*w*/*v*) sodium laurate.

To assess the membrane injury of Caco-2 cells caused by SLG-30, sodium laurate, and NaGC, the LDH activities were measured right after the transport studies. The solutions were collected from the apical sides of wells and then centrifuged at 200× *g* for 7 min at 4 °C. The activity of the released LDH was measured in the supernatant (LDH_release_). As shown in Figure 6A, the activity of LDH released from Caco-2 cells did not increase after the treatment with SLG-30 as compared to that in the control group. In contrast, the activity of LDH released significantly increased after the treatment with 0.05% (*w*/*v*) sodium laurate. Neither 0.1% (*w*/*v*) nor 1% (*w*/*v*) of NaGC increased the release of LDH. These findings proposed that SLG-30 did not cause serious injury to the cell membranes. Figure 6B shows the relationship between the effects of SLG-30 and typical absorption enhancers on the CF permeability across Caco-2 cells on one side and their toxic effects on the other side. While the absorption-improving effect of SLG-30 is concentration-dependent, we found no serious injury to enteric membrane irrespective of SLG-30 concentrations.

### 3.5. Estimation of Claudin Proteins by Western Blotting

**Figure 7:**
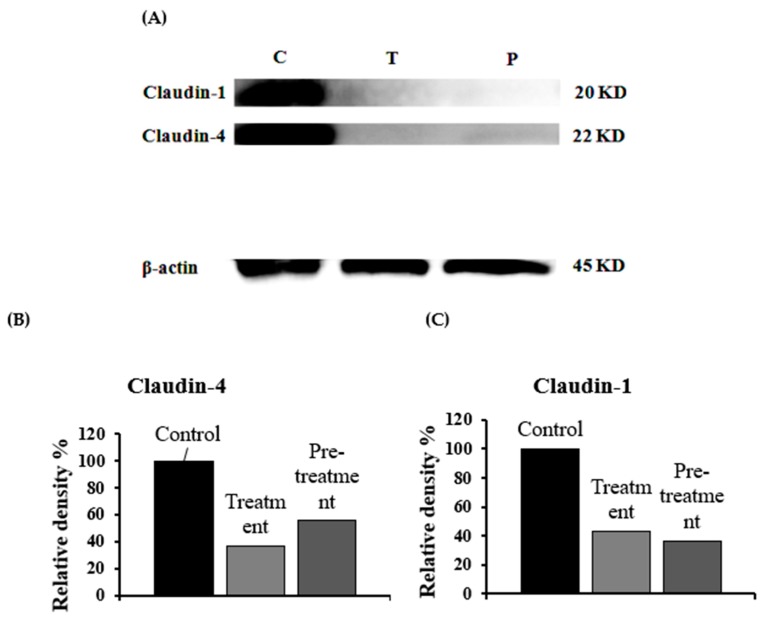
Western blot images of claudin-1 and claudin-4 (**A**): The expression levels of claudin-1 (**B**) and claudin-4 (**C**) in the rat small intestine were quantitatively calculated after a treatment with 0.5% (*v*/*v*) SLG-30 for 1 h. Control, without treatment; treatment, treatment with 0.5% (*v*/*v*) SLG-30 for 1 h; and pretreatment, pretreatment for 1 h. The proteins were extracted after 4 h.

As described above, SLG-30 might open the tight junctions of the intestinal epithelium, thus increasing the movement of substances through the paracellular route. However, the mechanism by which SLG-30 might open the tight junctions was not elucidated. Therefore, we finally evaluated the expression levels of claudin-1 and claudin-4 by western blotting. Figure 7A shows the bands of western blotting for claudin-1 and claudin-4 after a treatment with 0.5% (*v*/*v*) SLG-30. The treatment with 0.5% (*v*/*v*) SLG-30 decreased the intensity of the claudin-1 and claudin-4 bands by 57% and 64%, respectively, as shown in Figure 7B,C. The expression levels of claudin-1 and claudin-4 were about 64% and 45% less than the control, respectively, after the pretreatment study. 

## 4. Discussion

SLG-30 is a gemini surfactant with good surface-active properties and it is different from typical surfactants of similar chain lengths [21]. In this study, the efficacy of SLG-30 in improving enteric absorption was compared with that of a typical absorption enhancer. We used 1% (*w*/*v*) NaGC, a typical bile salt, as a typical absorption enhancer. Previously, it was proposed that bile acids interact with cell membranes to form reverse micelles, which act as a channel to increase permeation [35]. On the other hand, previous reports demonstrated the inhibition of insulin hydrolysis in different mucosal homogenates of rats by 1% (*w*/*v*) NaGC (a protease inhibitor) [36]. As shown in Figure 2, 1% (*w*/*v*) NaGC improved the absorption of CF from the small intestine to a much lower range than SLG-30. As shown in Table 1, the AUC_0→240_ value of CF in the presence of 0.5% *v*/*v* SLG-30 is 3.5 times greater than that in the presence of 1% (*w*/*v*) NaGC. These findings suggested that the absorption-enhancing effect of SLG-30, a gemini surfactant, is more potential than typical absorption enhancers.

We also studied the absorption-improving effect of sodium laurate, as a typical absorption enhancer and a major component of SLG-30 molecule. From Figure 2, we conclude that the effect of sodium laurate on the enteric absorption of CF was quite low and rapid, probably because sodium laurate itself was absorbed into the bloodstream due to its low molecular weight (approximately 288 Da), leading to a decrease in the concentration of sodium laurate at the site of absorption. This could explain the lower T_max_ observed for sodium laurate than for SLG-30. Table 1 also shows the difference between the AUC_0→240_ values of CF in the presence of SLG-30 and sodium laurate. Therefore, SLG-30 by itself, rather than sodium laurate, a degradation product of SLG-30, could improve the enteric absorption of poorly absorbable substances. These results provided three outcomes: firstly, the superiority of the enteric absorption-enhancing effects SLG-30 over typical absorption enhancers. Secondly, SLG-30 was effective as a whole compound and not because of lauric acid moieties present in the SLG-30 molecule. Finally, SLG-30 was stable in the intestines and was not degraded to its basic components. 

In this study, we examined and elucidated the absorption-enhancing mechanisms of SLG-30 in improving the enteric absorption of poorly absorbed substances. Of all the absorption-enhancing mechanisms, an increase in the membrane fluidity is one of the most important mechanisms of action of many absorption enhancers. Labeling the cell membrane with fluorescent compounds allows the tracking of membrane fluidity changes by fluorescence polarization techniques [25,28]. Enteric BBMVs were used to study the membrane lipid fluidity in the presence of SLG-30. This was done by measuring the fluorescence intensities and calculating the fluorescence anisotropies of DPH, tma-DPH, and DNS-Cl [37,38]. As shown in Figure 3A, SLG-30 slightly decreased the anisotropy of DPH; meanwhile, it decreased the anisotropy of DNS-Cl in a dose-dependent pattern, as shown in Figure 3C. These results suggested that SLG-30 has a great effect on the protein portion of the cell membrane with a mild effect on the inner portion between the phospholipids bilayers. However, it did not have an effect on the outer portion of the phospholipid bilayers, as indicated in Figure 3B.

Caco-2 cell monolayers were used in this study to understand the mechanism by which SLG-30 could improve the absorption of chemical compounds and peptides. Caco-2 monolayers are more susceptible to the cytotoxic influences of permeation enhancers than whole enteric tissue [39,40]. For this reason, we used low concentrations of SLG-30, i.e., 0.0001% (*v*/*v*), 0.001% (*v*/*v*), 0.025% (*v*/*v*), 0.05% (*v*/*v*), and 0.1% (*v*/*v*), and a low concentration of CF (10 µM). When SLG-30 was added at concentrations used in *in vivo* studies, the cells were detached and severely injured. The TEER values usually decreased when the tight junctions between the adjacent cells were opened. In the present study, SLG-30 at all concentrations, significantly decreased the TEER values, suggesting that this enhancer might open the tight junctions, even at low concentrations, thereby increasing the transport of CF. The TEER values did not recover to the baseline when high concentrations of SLG-30 were used, while in the *in vivo* absorption studies, we noticed that the concentrations of CF in the plasma decreased [22]. This distinction might be explained by the nature of Caco-2 cells, which are more sensitive than the enteric cells and have a lower ability to remove any substance away from the cell surface than in vivo enteric cells [12,40].

In the present work, we also compared the effects of typical absorption enhancers and SLG-30 on Caco-2 cells. As shown in Figure 5B, the effects of NaGC were consistent with the results of previous report [24] and with the *in vivo* studies. NaGC at a concentration of 1% (*w*/*v*) might also open the tight junctions to an extent lower than SLG-30. The cumulative amounts of CF (Figure 7) after using 1% (*w*/*v*) NaGC was also less than that obtained after using 0.001% (*v*/*v*) of SLG-30, thus confirming the superiority of SLG-30 over NaGC. Figure 4B also indicates that sodium laurate significantly decreased the TEER values without recovering and, with a significant cumulative amount of CF (Figure 5), even more than that of 0.1% (*v*/*v*) SLG-30, while in the in vivo studies, the absorption enhancement ratio for SLG-30 was better than that of sodium laurate. This discrepancy can be partially explained by the highly toxicity effects of sodium laurate on Caco-2 cells. As Figure 6A shows, the amount of LDH released from Caco-2 cells was significant, and sodium laurate might cause a disintegration of Caco-2 cell monolayers and a rupture of cell membranes which led to a high permeability of CF to the basal side.

We previously reported the *in vivo* safety of SLG-30, as an absorption enhancer for the enteric epithelium [22]. The results of toxicity studies on Caco-2 cells (Figure 6A) also confirm the safety of SLG-30 on the intestinal epithelium. Apparently, SLG-30 might loosen the tight junctions to an extent enough for drug permeability but not enough to cause cell detachment, as in the case of sodium laurate, or to cause damage to the cell membrane, as suggested by the normal release of LDH. 

Many previous studies have suggested the importance of claudin family proteins (molecular masses of approx. 23 kDa) to tight junctions to function properly [41,42]. Occludin family proteins are also important tight junction components. However, the deletion of occludin did not cause a disturbance of the TJs barrier function [43], and whether occludin is a major part of TJs remains unclear. Claudins, which have molecular weight of approx. 23 kDa, comprise a multigene family consisting of more than 20 members. In our experiment, we studied the levels of claudin-1 and claudin-4 after treatment with 0.5% (*v*/*v*) SLG-30. The expression levels of both claudin-1 and claudin-4 were impaired (Figure 7A), suggesting that the loosening of TJs might be connected to the decrease in the levels of these proteins and that SLG-30 increased enteric transport of substances through the paracellular route. The pretreatment studies indicated that these proteins levels recovered, although at different rates, which could explain the partially reversible effects of SLG-30 [22]. It seems that the tight junctions might need more than 4 h for recovery. However, other proteins involved in TJs should be tested, including occludin and ZO-1, and further studies are required to understand the underlying mechanism of the decrease in these protein levels.

These findings also suggested that SLG-30 might enhance the enteric absorption of substances through a paracellular route in addition to a transcellular route. 

## 5. Conclusions

Comparative studies conducted *in vivo* and *in vitro* showed that SLG-30, a gemini surfactant, had a greater absorption-improving effect than typical absorption enhancers, including NaGC and sodium laurate. Furthermore, the study also suggested that SLG-30 was stable in the intestines and acted as a whole compound to ameliorate the enteric absorption of poorly absorbed substances. The *in vitro* evaluation of membrane damage results confirmed the safety of SLG-30 on the enteric membrane. SLG-30 might also improve absorption through the transcellular route by changing the membrane fluidity. In addition, *in vitro* mechanistic studies suggested that SLG-30 might affect tight junctions by decreasing the levels of claudin-1 and claudin-4; thus, SLG-30 might open TJs and enhance the permeability through the paracellular route.

## Figures and Tables

**Table 1:** A summary of the pharmacokinetic parameters of CF (0.5 mg/kg) after its administration with 0.5% (*v*/*v*) SLG-30, 0.5% (*w*/*v*) sodium laurate, and 1% (*w*/*v*) sodium glycocholate into rat small intestines by an in situ closed-loop method.

Absorption Enhancer	Conc.	C_max_ (ng/mL)	T_max_ (min)	AUC_0__→240_ (ng·min/mL)	Enhancement Ratio
Control		-	-	5450 ± 700	-
SLG-30	0.5% (*v*/*v*)	487 ± 4	180 ± 0	87,200 ± 6000 **	16.0
Sodium laurate	0.5% (*w*/*v*)	375 ± 2	15 ± 0	14,700 ± 1950	2.7
Sodium glycocholate	1% (*w*/*v*)	172 ± 2	180 ± 0	24,500 ± 3700 *	4.5

The results are expressed as the mean ± S.E. (*n* = 3). ** *p* < 0.01, * *p* < 0.05, when compared with the control group.
